# Lymphatic vascular aging and age-related bone loss: current status and future perspectives

**DOI:** 10.3389/fimmu.2026.1900206

**Published:** 2026-09-01

**Authors:** Maryam Chudhary, Shuting Zhang, Pavel Docshin, Jing Liu, Tamara Aripova, Jiayin Zhou, Jinghao Li, Anna Malashicheva, Ju Liu

**Affiliations:** 1 Institute of Microvascular Medicine, The First Affiliated Hospital of Shandong First Medical University & Shandong Provincial Qianfoshan Hospital, Jinan, China; 2 Shandong First Medical University, Jinan, China; 3 Medical Research Center, Shandong Provincial Qianfoshan Hospital, Jinan, Shandong, China; 4 School of Traditional Chinese Medicine, Shandong University of Traditional Chinese Medicine, Jinan, China; 5 Laboratory of Regenerative Biomedicine, Institute of Cytology Russian Academy of Science, St. Petersburg, Russia; 6 Shandong Provincial Key Medical and Health Laboratory of Translational Medicine in Microvascular Aging, Jinan, China; 7 Laboratory for Future Industry in Gene Editing in Vascular Endothelial Cells of Universities of Shandong Province, Jinan, China; 8 Institute of Immunology and Human Genomics, Academy of Sciences of Uzbekistan, Tashkent, Uzbekistan

**Keywords:** age-related bone loss, lymphangiogenesis, lymphatic vascular aging, osteoimmunology, osteoporosis

## Abstract

Age-related bone loss is a major contributor to osteoporosis and fragility fractures in older adults. Skeletal aging is accompanied by reduced bone mineral density, impaired bone microarchitecture, chronic low-grade inflammation, and immune dysregulation. Lymphatic vascular aging refers to age-related structural and functional decline of lymphatic vessels. This decline impairs immune surveillance and inflammatory mediator clearance, thereby disrupting tissue homeostasis. Age-related lymphatic dysfunction impairs drainage and inflammatory clearance. This allows inflammatory mediators, including IL-6 and TNF-α, to persist in bone-associated tissues, thereby promoting osteoclastogenesis and resorption-dominant remodeling. Age-related lymphatic dysfunction also affects VEGF-C/VEGFR-3 signaling, chemokine-mediated immune trafficking, and marrow niche support. These changes link drainage failure to osteoimmune imbalance and delayed bone repair. Current evidence supports a link between lymphatic vascular dysfunction and skeletal degeneration. Most evidence comes from animal models, bone injury studies, or diseases with secondary lymphatic defects, whereas direct clinical evidence in human age-related osteoporosis remains limited. This review summarizes current evidence linking lymphatic dysfunction to skeletal degeneration, examines the context-dependent roles of lymphatic remodeling in skeletal homeostasis, and discusses emerging therapeutic strategies targeting the lymphatic-bone axis. Future studies should define clinically relevant lymphatic alterations and determine whether restoring lymphatic homeostasis can mitigate age-related bone loss.

## 1 Introduction

Age-related bone loss is a common skeletal disorder that leads to skeletal fragility, impaired fracture repair, and disability in older adults. Recent GBD 2021 data further show that fractures attributable to low bone mineral density remain a major source of global morbidity and mortality (1). About one in three women and one in five men over age 50 will experience an osteoporotic fracture during their lifetime (2). Aging induces cellular senescence in bone, disrupting bone remodeling and altering the balance between osteoclast-mediated resorption and osteoblast-driven formation (3). With age, senescent cells accumulate and develop a senescence-associated secretory phenotype (SASP) that releases inflammatory cytokines, disrupting bone homeostasis (4–6). Specifically, osteocytes, osteoblast lineage cells, and bone marrow stromal cells gradually age, contributing to cortical porosity, trabecular thinning, and impaired bone regeneration (7, 8). Currently, anti-resorptive and anabolic therapies reduce fracture risk in older adults; however, these treatments primarily target bone remodeling rather than mechanisms contributing to bone aging (9–11).

The lymphatic vasculature regulates drainage, immune cell trafficking, and inflammatory clearance in tissues linked to skeletal homeostasis. The lymphatic network clears cytokines and facilitates immune cell egress, thereby supporting bone homeostasis and the bone marrow niche (12, 13). With age, however, lymphatic vessel function declines, leading to impaired cytokine clearance, reduced immune surveillance, and disruption of the bone microenvironment (14–16). A key preclinical study reported lymphatic vessels within bone and linked their expansion to bone regeneration following injury (17). Additional evidence from developmental, disease, and injury models further supports the presence of intraosseous lymphatic vessels and their potential roles in immune regulation and tissue repair (13, 18, 19). However, their precise anatomical distribution across bone compartments and their physiological functions under normal conditions remain incompletely defined. More broadly, studies of lymphatic vascular aging and inflammaging associate impaired lymphatic function with inflammatory retention, delayed tissue repair, and skeletal decline (20, 21). In parallel, defective lymphatic drainage intensifies osteoclastogenic signaling and disrupts the functional coordination between osteoblasts and osteoclasts (22, 23). Current studies suggest that age-related lymphatic dysfunction may contribute to inflammatory bone loss through drainage failure, cytokine retention, and osteoimmune imbalance (24, 25). Accordingly, this review discusses these downstream mechanisms based on the available evidence, distinguishing established findings from preclinical observations and proposed links to common age-related bone loss.

The term lymphatic aging refers to age-related changes in the lymphatic system. We discuss lymphatic aging at both the vessel level, including structural and functional changes such as impaired lymphatic drainage and reduced contractility, and the cellular level, including LEC dysfunction and cellular senescence as a cellular-level concept. The resulting age-related lymphatic dysfunction contributes to osteoimmune imbalance and age-related bone loss.

Despite extensive research on age-related bone loss, the role of lymphatic dysfunction in skeletal degeneration remains insufficiently understood. Direct evidence in human skeletal aging remains limited. Evidence from rare lymphatic-bone disorders, inflammatory bone diseases, and secondary lymphatic injury provides mechanistic clues, but these settings only partially reflect the slow and multifactorial course of common senile osteoporosis. Clarifying how lymphatic dysfunction contributes to osteoimmune imbalance and skeletal aging is essential for identifying novel therapeutic targets and improving strategies to prevent age-related bone loss.

In this review, we summarize evidence linking lymphatic vascular aging to skeletal degeneration, focusing on age-related lymphatic dysfunction, including drainage failure, inflammatory retention, osteoimmune imbalance, and repair-associated lymphangiogenesis. We further distinguish repair-associated lymphangiogenesis from pathological lymphatic expansion, highlighting how the biological context determines their effects on skeletal homeostasis and age-related bone loss.

## 2 Lymphatic and skeletal interplay during aging

**Figure 1:**
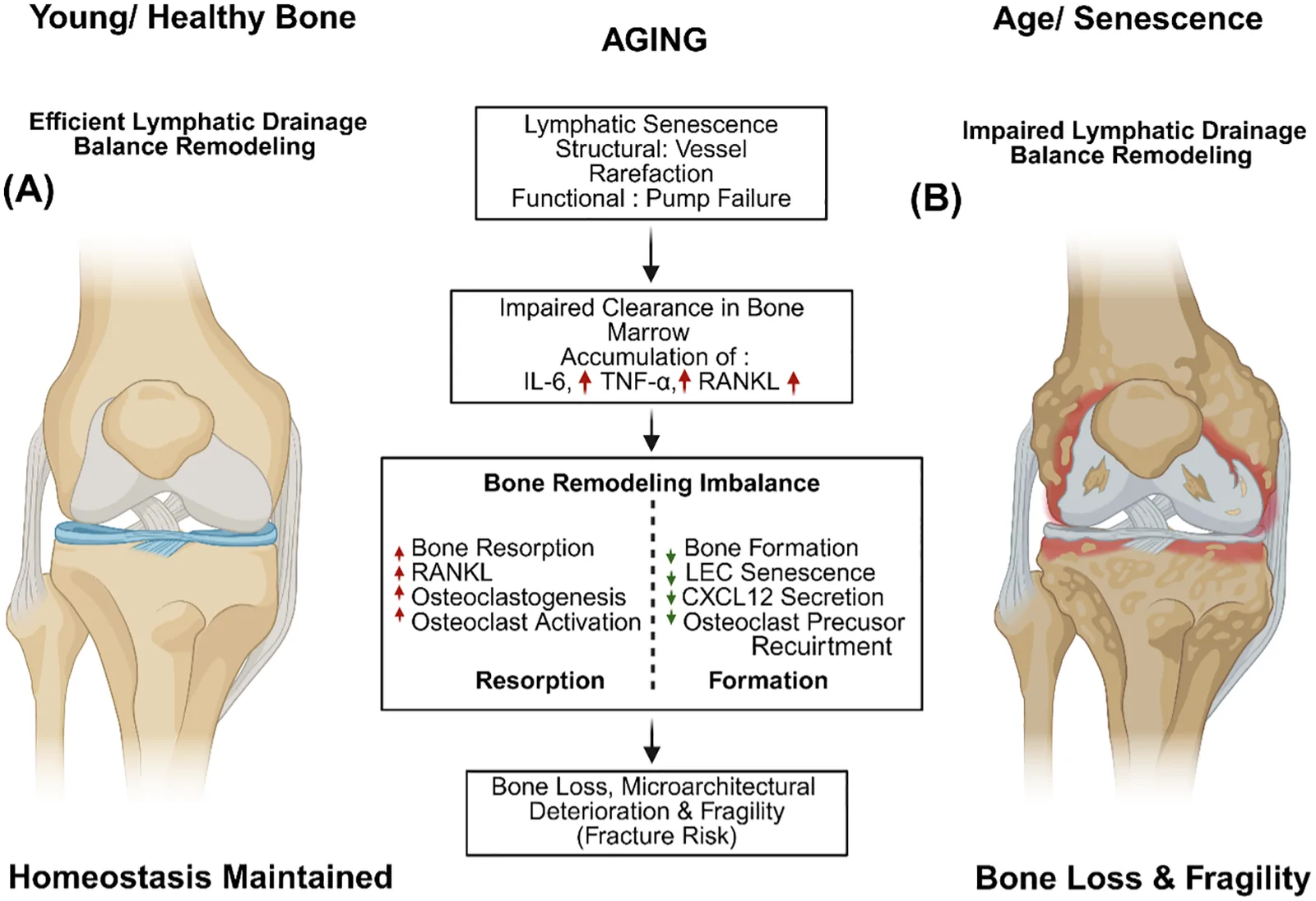
Aging-associated lymphatic senescence disrupts bone remodeling and promotes skeletal fragility. **(A)** In young, healthy bone, efficient lymphatic drainage maintains tissue fluid balance, clears inflammatory mediators, and supports coordinated bone remodeling, thereby preserving skeletal homeostasis. **(B)** With aging, reduced lymphatic function may impair inflammatory clearance and favor resorption-dominant remodeling. These proposed relationships are based mainly on preclinical evidence, and the contribution of functional intraosseous lymphatics to physiological skeletal aging remains to be established. Created in BioRender. Ahmed, N. (2026) https://BioRender.com/tmoh2ed.

Understanding lymphatic-bone crosstalk is essential for identifying the drivers of their coordinated decline with age. This section highlights key changes in lymphatic architecture and skeletal integrity that accelerate fragility. Bone mass progressively declines after age 40, increasing structural vulnerability and fracture risk (7). Preclinical evidence identifies lymphatic dysfunction as a contributor to skeletal fragility, highlighting the need for systemic interventions that restore both vascular and skeletal homeostasis (26). An overview of how lymphatic vascular aging contributes to impaired lymphatic drainage and age-related bone loss is presented in **Figure 1**.

### 2.1 Lymphatic vascular aging

Lymphatic vascular aging is characterized by age-related structural and functional changes in lymphatic vessels, including matrix degradation, elastin fragmentation, smooth muscle loss, and glycocalyx depletion (8, 27, 28). These vessel-level aging changes should be distinguished from bona fide cellular senescence of LECs, which requires direct senescence-marker evidence. Structural changes in aged lymphatic vasculature, including matrix remodeling, elastin fragmentation, and microcalcification, increase vessel stiffness and restrict unidirectional flow (8, 10). In addition, these age-related mechanical changes further destabilize endothelial junctions, thereby promoting immune cell infiltration and sustained inflammation in aged tissues (12, 29).

Lymphatic endothelial cell (LEC) dysfunction is a central feature of lymphatic aging. In aged tissues, LEC impairment manifests as reduced contractility, weakened barrier integrity, and impaired immune clearance in both human and *in vivo* models (14, 30). Consistent with this, vessel density and contraction frequency decline with age, leading to pump failure and lymphatic reflux (14, 15). Aged or dysfunctional LECs have been associated with altered redox balance, increased susceptibility to oxidative stress, and reduced adaptive lymphangiogenic responses during aging (29). In aged rats, for instance, mast cell-NF-κB signaling increases permeability and reduces contractility in mesenteric lymphatics (25, 31). Moreover, dysregulated peri-lymphatic mast cells further impair lipid uptake and immune cell trafficking in age-associated lymphedema (32).

Lymphangiogenic failure further disrupts structural renewal and immune cell trafficking (33). In aged tissues, downregulation of VEGF-C/VEGFR-3 signaling and macrophage dysfunction destabilize vascular remodeling and impair lymphatic maintenance (33, 34). In addition, VEGF-C/VEGFR-3 downregulation in aged lymphatics reduces immune cell trafficking by approximately 40-60%, thereby disrupting tissue homeostasis and impairing inflammatory resolution (34, 35). However, the mechanisms driving lymphangiogenic decline in aging lymphatic vessels remain incompletely understood. Targeting inflammatory signaling may represent a promising strategy to restore lymphatic immune plasticity and preserve skeletal homeostasis during aging; however, its efficacy in human lymphatic aging remains to be validated.

### 2.2 Lymphatic vascular aging in bone microenvironment

Within bone-associated tissues, lymphatic vessels have been proposed to facilitate fluid drainage, inflammatory mediator clearance, and immune cell egress (13). However, the extent to which age-related lymphatic dysfunction contributes to skeletal homeostasis remains incompletely defined (13, 36). Available experimental evidence suggests that impaired lymphatic transport may reduce fluid clearance, promote inflammatory retention, and alter bone remodeling, although these observations remain largely limited to preclinical models (37, 38).

Downregulation of lymphatic-derived chemokines may limit immune egress and disturbs osteoimmune coupling (22, 39, 40). Loss of adequate lymphatic clearance and accumulation of inflammatory mediators contribute to skeletal fragility and increase fracture risk in the elderly (17, 38). In addition, loss of VEGF-C/VEGFR-3 signaling reduces remodeling and clearance capacity, exacerbating interstitial inflammation and osteoclastogenic activity *in vivo* (41, 42). Reduced lymphangiogenesis, along with impaired shear stress-mediated mechanotransduction, further limits lymphatic fluid clearance, thereby intensifying interstitial inflammation in aged tissues (5, 43). Persistent SASP-associated inflammation prolongs lymphatic dysfunction and sustains a proinflammatory microenvironment that favors osteoclast activation in the elderly (6, 8, 44, 45). Collectively, these findings suggest that impaired lymphatic clearance may promote a pro-resorptive microenvironment during aging, although direct evidence in humans remains limited (38, 46).

Clinical observations support an association between inflammatory imbalance and skeletal decline. Circulating inflammatory markers, including IL-6, TNF-α, and CRP, are persistently elevated in aged individuals and inversely correlate with BMD and fracture risk across multiple cohorts (23, 24, 47, 48). Elevated IL-6 also correlates with increased bone turnover markers, such as β-CTX, indicating an imbalance in active remodeling (49). However, direct clinical evidence linking aged lymphatic failure to human bone disorders remains limited (20).

### 2.3 Lymphatic vascular aging in the marrow osteoimmune niche

Aging markedly exacerbates lymphatic dysfunction within the bone marrow, increasing the risk of osteolysis and inflammaging in elderly individuals (20, 50). Senescent LECs lose their ability to sustain immune homeostasis, resulting in CXCL12^+^ MSC depletion, osteoblast insufficiency, and myeloid skewing (39, 51, 52). Immunosenescence further reduces lymphoid progenitor output and plasma cell retention, while effector memory T cells are dysregulated, destabilizing the osteoimmune niche (53–55).

TNF-α-mediated suppression of CXCL12 compromises plasma cell survival. Similarly, increased reactive oxygen species (ROS) accumulation in CXCR4-deficient stromal cells promotes HSC dysfunction and myeloid bias in aged mice (56–58). Consistent with this, conditional deletion of CXCL12/SDF-1 in stromal cells disrupts HSC retention and lymphoid progenitor support *in vivo*, underscoring the importance of CXCL12-dependent niche maintenance (59, 60). In aged bone marrow, mesenchymal stromal cells exhibit reduced CXCR4 expression and increased ROS production, thereby impairing niche-supporting activity and promoting hematopoietic stem and progenitor cell defects (58). Additional inflammatory pathways, including macrophage-associated APP–CD74 signaling and VCAM1-mediated lymphocyte recruitment, sustain a proinflammatory state, exacerbating osteoimmune disruption (61, 62). IFN-γ and IL-6 further reinforce niche inflammation by maintaining senescent CD8^+^CD28^−^ T-cell phenotypes and suppressing factors that support plasma cell survival (63–65).

Lymphatic clearance deficits further exacerbate oxidative stress and weaken the osteoimmune interface, thereby increasing osteoporosis risk (58, 66, 67). In multiple myeloma, elevated CXCL12 is associated with osteoclast motility and bone resorption, while stromal insufficiency contributes to marrow failure syndromes (68, 69). Notably, aged marrow endothelial cells secrete excessive ROS, polarizing T cells toward CD8^+^CD28^−^ T phenotypes and perpetuating niche inflammation (70, 71).

## 3 Molecular mechanisms of lymphatic-bone crosstalk in aging

**Figure 2:**
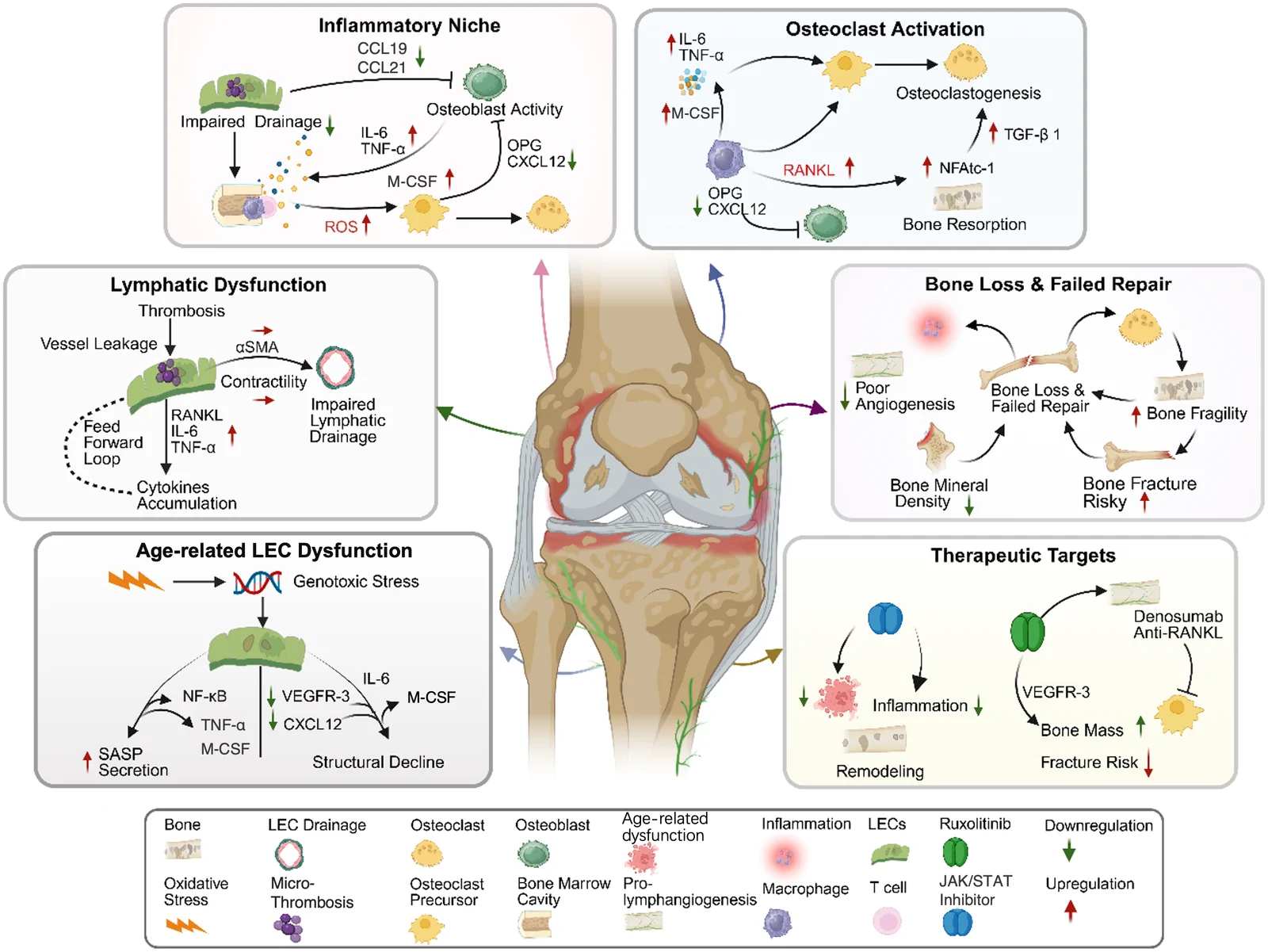
Experimental evidence and proposed mechanisms of age-related lymphatic dysfunction in bone aging. The process begins with age-related stress in lymphatic endothelial cells (LECs). Prolonged oxidative and genotoxic stress gradually drives these cells into senescence, and as this occurs, their normal regulatory functions decline. NF-κB signaling remains active for prolonged periods, leading to sustained release of inflammatory mediators, including IL-6, TNF-α, and M-CSF. At the same time, expression of VEGFR-3 and CXCL12 declines, weakening lymphatic stability. Reduced αSMA levels and impaired vessel contractility lead to leakage, thrombosis, and poor lymphatic drainage. Persistent oxidative stress disrupts tissue balance by increasing reactive oxygen species, enhancing nuclear factor of activated T cells 1 (NFATc1) activity, and shifting the OPG/RANKL ratio toward RANKL dominance, thereby promoting osteoclast differentiation and sustained resorption. Over time, bone breakdown surpasses bone formation. Osteoblast activity is insufficient to counteract this loss; trabecular structures become thinner and less interconnected, and BMD decreases, compromising mechanical strength. Vascular responses also appear inadequate. Angiogenesis does not fully compensate for ongoing damage, and repair mechanisms remain limited. As regeneration falls short, microstructural defects accumulate, potentially explaining the progressive increase in fracture risk. Therapeutically, JAK/STAT inhibition may reduce SASP-associated inflammatory signaling, whereas VEGF-C-based approaches may enhance VEGFR-3-dependent lymphatic remodeling and drainage in preclinical models. Evidence-supported elements shown in this figure include impaired lymphatic drainage and contractility, inflammatory mediator retention, ROS/NF-κB-associated inflammatory signaling, VEGF-C/VEGFR-3-dependent lymphatic remodeling during repair, and CXCL12-associated marrow niche support reported in preclinical models. Proposed links include direct LEC senescence-related SASP effects and their causal contribution to common age-related osteoporosis, which require validation with LEC-specific senescence markers and human skeletal aging data. Created in BioRender. Ahmed, N. (2026) https://BioRender.com/909mi6v.

Aging-associated lymphatic dysfunction alters inflammatory signaling, redox balance, and immune regulation within the skeletal microenvironment. These molecular disturbances disrupt osteoimmune homeostasis and promote bone resorption. The key molecular pathways by which age-related lymphatic dysfunction contributes to bone aging are summarized in **Figure 2**.

### 3.1 Drainage failure-driven osteoclast activation

Lymphatic drainage maintains fluid balance and immune stability in bone and regulates skeletal homeostasis throughout life (72). Human imaging studies and experimental aging models show that lymphatic vessel density declines in musculoskeletal tissues with age, reducing lymph transport capacity (8, 73). Aging also weakens the collecting lymphatic vessels. In aged mice, these vessels lose smooth muscle coverage and contract less efficiently, thereby reducing lymph flow and limiting tissue drainage (12).

Reduced lymphatic transport impairs the clearance of inflammatory mediators from the bone microenvironment, thereby promoting inflammatory signaling (20, 74). Elevated cytokine levels increase RANKL expression and stimulate osteoclast precursor differentiation in aged bone (20, 74). Persistent inflammatory signaling, therefore, shifts bone remodeling toward resorption. Aged murine models further show that lymphatic rarefaction disrupts the OPG/RANK/RANKL signaling axis and increases osteoclast formation, accelerating cortical bone erosion (36).

Evidence from inflammatory bone conditions further supports these structural changes. Fracture and periodontitis models show expansion of LYVE1^+^ lymphatic capillaries, accompanied by a reduction in PDPN^+^/αSMA^+^ collecting vessels surrounding the alveolar bone (72). These alterations impair lymphatic transport capacity and limit the efficient clearance of interstitial fluid and inflammatory mediators (5, 72). Moreover, age-related changes in tissues surrounding lymphatic vessels reduce vessel flexibility and further compromise lymphatic transport, contributing to cortical bone damage and porosity in elderly bone (73). Collectively, lymphatic drainage failure in aged bone may promote inflammatory retention and enhance osteoclast activation and migration. These changes shift remodeling toward bone resorption and skeletal fragility.

### 3.2 Oxidative stress-induced barrier and osteocyte dysfunction

Aged bone fragility is not limited to excessive bone resorption but also reflects compromised bone quality and weakened structural integrity (75). Oxidative stress and inflammation are major contributors to LEC dysfunction in aging tissues. Aged lymphatic vessels exhibit glycocalyx loss, smooth muscle injury, and extracellular matrix degradation, changes that weaken lymphatic barrier integrity near bone and marrow compartments (8). In addition, alterations in junctional proteins, including VE-cadherin, further increase lymphatic permeability adjacent to bone tissue (76). As the lymphatic barrier becomes less stable, lymphatic drainage becomes inefficient, and oxidative mediators accumulate in the skeletal microenvironment.

Accumulation of reactive oxygen species further disrupts lymphatic transport in aged tissues. Superoxide radicals, mitochondrial ROS, and thiobarbituric acid-reactive substances impair lymphatic contractile function and reduce drainage efficiency (5, 25). Most importantly, reduced lymphatic clearance allows oxidative mediators to spread from lymphatic sources into the skeletal compartment (77, 78). The elevated oxidative environment directly affects bone cells. ROS, generated by NADPH oxidase 4 (NOX4), promote osteoclast differentiation and survival, thereby increasing bone resorption (77, 78). As evidence, NOX4-deficient mice show increased BMD and decreased osteoclast numbers, supporting the role of NOX4-derived ROS in skeletal resorption (77).

Furthermore, in aging bone, ROS-induced lymphatic dysfunction destabilizes the bone marrow microenvironment and impairs the osteoimmune interface (58, 66, 67). Elevated ROS levels promote T-cell senescence, hematopoietic stem cell dysfunction, and myeloid bias (56, 57, 70, 71), thereby disrupting the osteoimmune interface and compromising immune homeostasis within the bone marrow microenvironment (70, 71). Oxidative stress also compromises osteocyte viability and bone structural integrity. Increased oxidative stress promotes mast cell degranulation and stimulates the release of VEGF and IL-6, thereby amplifying inflammatory signaling (8, 79). Moreover, ROS-induced genotoxic stress, an important contributor to oxidative damage, fails to elicit an adequate pro-lymphangiogenic response in aged LECs, affecting bone and hematopoietic renewal after injury (8). Chronic ROS and SASP exposure cause DNA damage in osteocytes, triggering p16/p21 signaling and ultimately leading to osteocyte senescence or apoptosis (80). Loss of these osteocytes disrupts the lacunocanalicular network and compromises mechanosensory and nutrient transport functions, thereby contributing to cortical porosity and skeletal fragility (80, 81).

### 3.3 SASP-associated inflammation and remodeling imbalance

Aging-induced bone loss is not merely due to enhanced bone resorption; it is also associated with progressive changes in the quality of bone remodeling (80). Cellular senescence increases with age in several skeletal cell populations, including osteocytes, osteoblast-lineage cells, and marrow stromal cells. These cells release SASP-related mediators that contribute to chronic inflammation in aged bone. Under normal conditions, lymphatic drainage removes inflammatory mediators from bone-associated tissues, including periosteal regions (8, 13, 17). With age, impaired lymphatic transport may compromise this clearance function, allowing SASP-related and other inflammatory mediators to sustain within the bone microenvironment. Evidence for LEC senescence has been reported in selected experimental settings, but most skeletal aging studies do not directly validate LEC senescence using p16^INK4a/p21^CIP1 expression, SA-β-gal activity, or growth-arrest markers.

SASP-associated cytokines derived from skeletal cells impair immune surveillance, suppress lymphangiogenesis, and prolong tissue repair deficits in aged lymphatic tissues (21, 22). In aged tissue, impaired lymphatic clearance allows these mediators to persist in periosteal and marrow tissues, promoting inflammatory signaling (8, 13). In aged or dysfunctional lymphatic tissues, increased ROS can activate NF-κB signaling and promote the production of TNF-α and IL-6, two inflammatory mediators involved in SASP-related responses (10, 37, 82). ROS-dependent NF-κB activation further amplifies cytokine production and sustains a local inflammatory feedback loop (31). These inflammatory signals increase RANKL- and M-CSF-associated osteoclastogenic activity and accelerate trabecular bone loss (37, 78).

The presence of senescent cells reduces the efficiency of bone repair and fracture healing (80, 83). In aged bone, senescent stromal cells acquire SASP and impair bone repair efficiency (42, 83). In SAMP6 mice, an accelerated-aging model, senescent mesenchymal stem cells lose osteogenic potential and accelerate age-related bone loss (83, 84).

Oxidative stress further amplifies SASP-associated inflammatory signaling in aged lymphatic tissues. Antioxidant depletion, together with elevated ROS, activates NF-κB signaling and promotes cytokine cascades that disrupt skeletal homeostasis (9, 45, 49). In aged rats, mast cell-NF-κB signaling also increases lymphatic permeability and reduces lymphatic contractility in mesenteric lymphatics (25, 31). Aging-associated dysregulation of peri-lymphatic mast cells further contributes to lymphatic dysfunction and impaired local immune responses (32). Together, impaired lymphatic clearance and persistent inflammatory signaling may favor accumulation of mediators such as IL-6 and TNF-α in bone-associated tissues. These mediators can originate from stromal cells, immune cells, osteocytes, and dysfunctional lymphatic endothelium. Their persistence favors resorption-dominant remodeling and weakens trabecular bone integrity in aging populations (6, 8, 44, 45). Changes in the bone matrix associated with chronic SASP exposure may further compromise bone material properties, although the underlying mechanisms remain incompletely understood.

### 3.4 Immune trafficking and osteoimmune imbalance

Bone homeostasis depends on coordinated immune cell turnover in the marrow and endosteal niches; this process is progressively impaired with aging (22). The lymphatic vasculature supports this balance by regulating immune cell egress and chemokine-mediated trafficking between bone and lymphoid organs (13). Efficient lymphatic transport is therefore important within the skeletal microenvironment. However, aging progressively weakens lymphatic function and disrupts immune regulation in bone.

With aging, the marrow microenvironment develops impaired immune homeostasis and stromal decline; aged or dysfunctional LECs lose the ability to maintain immune equilibrium (13, 22, 83). The imbalance contributes to the depletion of CXCL12^+^ mesenchymal stem cells, reduced osteoblastogenesis, and a shift toward myeloid-biased differentiation within aged marrow (39, 51, 52). At the same time, decreased production of lymphatic-derived chemokines such as CCL19 and CCL21 limits immune cell egress from skeletal tissues (22, 39, 40). Reduced immune trafficking weakens osteoblast-osteoclast coupling and favors increased bone resorption.

Aging-associated lymphatic impairment also alters cytokine profiles and T-cell subsets in aged bone (48). Senescent CD4^+^CD28^−^ and CD8^+^CD28^−^ T cells produce osteoclast-promoting mediators, including RANKL, TNF-α, and IL-17, thereby sustaining osteoclast activation (42, 85). Persistent IFN-γ-mediated T-cell responses further regulate RANKL-dependent osteoclastogenesis and shift the OPG/RANK/RANKL axis toward bone resorption (85, 86).

Chemokine signaling also links lymphatic dysfunction to impaired skeletal regeneration. LECs produce CXCL12, a key regulator of bone formation and perivascular niche support (13, 18). In the intraosseous injury model reported by Biswas et al., LEC-derived CXCL12 promoted expansion of Myh11^+^CXCR4^+^ pericytes and supported skeletal regeneration (17). Following lymphatic injury, CXCL12 levels decline, and bone formation decreases. This specific lymphangiocrine axis remains mainly supported by preclinical intraosseous injury data and requires validation in additional aging and human skeletal models.

## 4 Lymphatic vascular dysfunction and impaired bone repair

**Figure 3:**
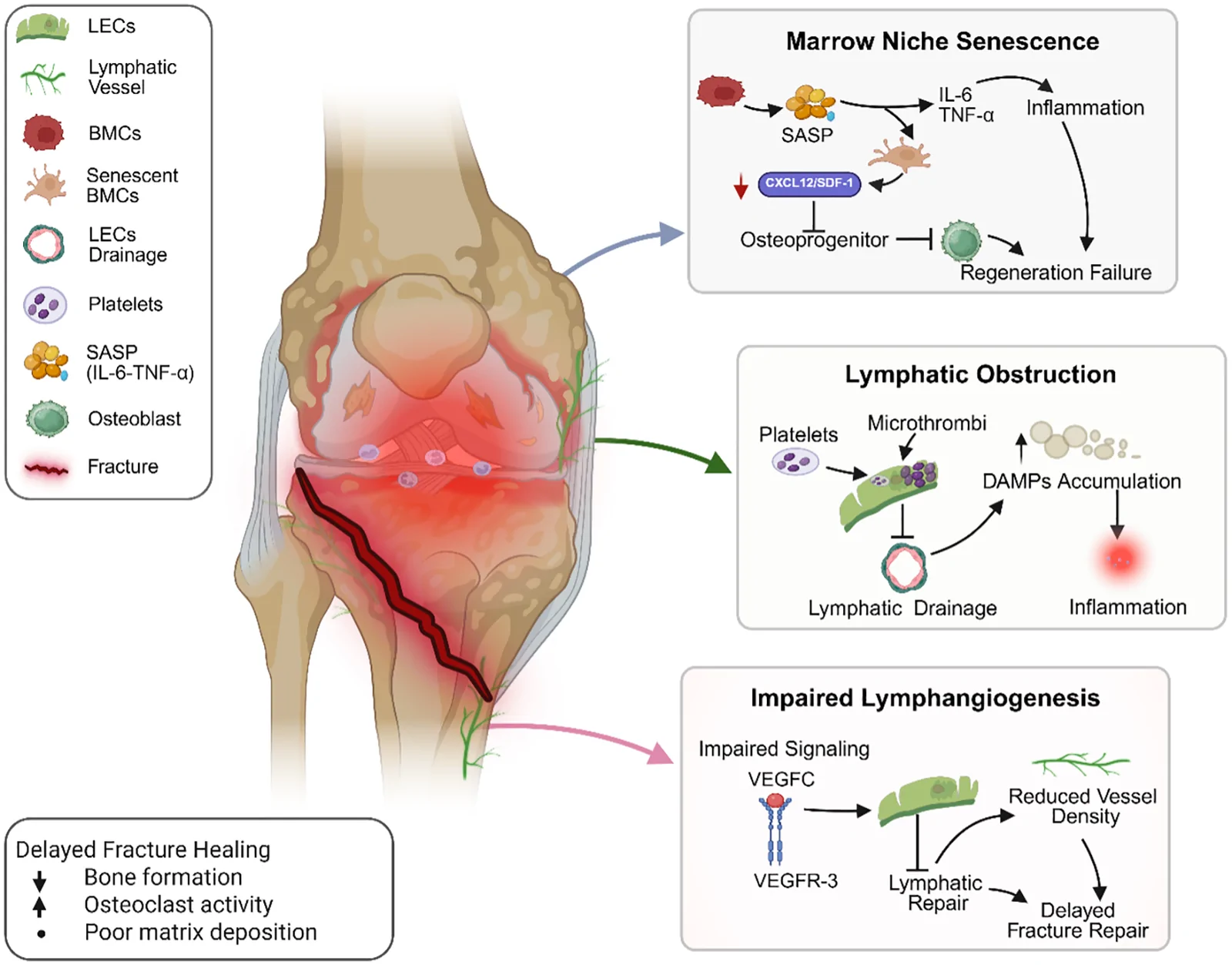
Lymphatic dysfunction impairs bone regeneration and fracture healing during aging. With aging, senescent stromal cells and other niche cells accumulate in the bone marrow, creating a SASP-associated proinflammatory environment that impairs skeletal repair. At the same time, reduced CXCL12 signaling weakens osteoprogenitor maintenance and retention, ultimately diminishing bone’s regenerative capacity. After a fracture, lymphatic function declines, with reduced vessel contractility and impaired drainage. Platelets aggregate within lymphatic vessels, forming microthrombi that impede normal vascular function. This reduction in lymphatic flow facilitates the accumulation of inflammatory mediators and DAMPs, exacerbating inflammation and interfering with osteogenic signaling pathways. In addition, impaired signaling through the VEGF-C/VEGFR-3 pathway limits the development of new lymphatic vessels, reduces lymphatic remodeling, and lowers vessel density. This compromises lymphatic repair, contributing to delayed fracture healing and the formation of a weak callus. Created in BioRender. Ahmed, N. (2026) https://BioRender.com/5vul8r0.

Lymphatic drainage supports the resolution of inflammation and bone repair. Age-related lymphatic dysfunction reduces drainage and promotes inflammatory retention in the marrow niche. As shown in **Figure 3**, these changes impair osteogenic signaling and delay fracture healing.

### 4.1 Bone marrow niche senescence and impaired regeneration

Effective bone repair depends on a functional bone marrow niche that maintains the coordinated balance between osteogenic and hematopoietic cells throughout life (36). The healthy marrow supports osteoprogenitor survival and differentiation, whereas its decline with age delays skeletal regeneration and increases fracture risk (36). Chronic inflammation and cellular senescence destabilize stromal integrity and weaken the regenerative capacity of aged bone following injury (12, 36).

Recent preclinical studies have identified lymphatic vessels within bone repair environments; however, their contribution under physiological conditions remains under investigation (17, 87). Within the bone repair microenvironment, lymphatic vessels are proposed to facilitate local fluid drainage and inflammatory clearance that may support skeletal reparative process during bone healing (17, 87). In experimental bone injury models, VEGF-C/VEGFR-3-dependent lymphatic responses limit excessive osteoclast differentiation and osteolysis, thereby facilitating coordinated skeletal repair (42). However, lymphatic insufficiency significantly delays the bone repair process (88). Reduced lymphatic clearance allows inflammatory mediators and SASP factors to accumulate within the marrow niche, disrupting stromal support and delaying skeletal regeneration (8, 88). Persistent SASP signaling disrupts stromal cell function and interferes with lymphatic-assisted repair processes. As a result, senescent stromal cells impair the osteogenic capacity of bone marrow mesenchymal stem cells (BMSCs) and weaken bone-forming activity during regeneration (12, 42, 83). In murine aging models, including SAMP6 mice, aged mesenchymal stem cells exhibit diminished osteogenic differentiation, thereby accelerating skeletal decline and delaying bone regeneration (83, 84).

Chronic inflammation induces senescence in bone marrow stromal cells and mesenchymal progenitors. These senescent cells show reduced proliferative capacity and impaired differentiation potential, thereby limiting osteoprogenitor output during skeletal repair (89). Genetically modified model (p16Ink4a)- driven mast cell senescence further amplifies inflammatory pathways and worsens marrow health (90). At the same time, IL-6 contributes to LEC dysfunction in aged mice, disrupting communication between immune cells and stromal progenitors within the niche (20, 29). Together, these changes destabilize the stem cell microenvironment and delay bone regeneration.

Chemokine imbalance further contributes to the failure of bone regeneration in aged bone. Expression of CXCL12/SDF-1 declines in aged marrow, destabilizing the perivascular niche required for progenitor retention and osteogenic commitment (17, 60). Loss of coordinated CXCL12-mediated signaling weakens osteogenic progenitor recruitment during fracture repair and limits effective callus formation in aged fracture models (17). Restoring lymphatic function or inhibiting SASP signaling improves lymphatic flow and reduces bone loss in experimental systems (12, 88). Collectively, age-associated lymphatic dysfunction, stromal senescence, oxidative stress, and chemokine imbalance converge within the aged marrow niche to suppress osteoprogenitor output and impair bone repair in the elderly population.

### 4.2 Lymphatic obstruction during fracture healing

Efficient fracture healing requires timely resolution of tissue edema and inflammatory mediators to allow the transition from inflammation to repair, a process that becomes less efficient with aging (13, 45). Lymphatic vasculature is central to this process, as it removes excess interstitial fluid, inflammatory cytokines, and damage-associated molecular patterns (DAMPs) from the fracture environment (13, 45). However, lymphatic transport capacity declines in aged tissues, limiting clearance during fracture repair (13, 45).

Lymphatic deficiency disrupts early fracture healing. In male mice, post-fracture lymphatic abnormalities persist for at least two weeks, contributing to delayed callus formation and thereby disrupting the healing process (38). These abnormalities disrupt the microenvironment required for osteoblast activity and matrix formation.

Persistent lymphatic obstruction further worsens drainage failure in aged bone. Following a fracture, local inflammatory cues activate circulating platelets via P2Y12-dependent pathways that regulate platelet aggregation and thrombus formation, and platelets become hyperresponsive with aging (91). Activated platelets adhere to lymphatic microvessels and form microthrombi within injured lymphatic channels. These microthrombi prevent the efficient clearance of DAMPs and inflammatory mediators that normally promote regenerative signaling (91). Reduced lymph flow, therefore prolongs inflammatory signaling within the fracture environment, impairing drainage in aged inflammatory settings (91). In aged bone, platelet-mediated thrombosis, together with fibrin deposition, occludes lymphatic microvessels and reduces lymphatic transport capacity at the injury site (92). A decrease in lymphatic drainage promotes the accumulation of inflammatory cytokines, such as IL-6 and TNF-α, along with cellular debris and DAMPs within the fracture microenvironment (5, 20). Sustained inflammatory signaling interferes with osteoblast differentiation and disrupts matrix deposition at repair sites.

Imaging studies further support a link between lymphatic obstruction and defective fracture repair. Near-infrared indocyanine green (ICG) imaging shows persistent tracer retention in fracture tissues, restricted lymphatic flow, and delayed callus formation (8, 38). Clinical lymphoscintigraphy findings are summarized in Section 5.4.

Restoration of lymphatic flow improves fracture healing in preclinical studies. Pharmacological inhibition of platelet aggregation with clopidogrel, a P2Y_12_ inhibitor, restores lymphatic flow, improves vascular contractility, and supports osteogenic repair capacity in aged fracture models (92). Similarly, VEGF-C-mediated lymphangiogenic stimulation clears lymphatic thrombi and accelerates tissue recovery by restoring drainage efficiency (92).

Collectively, aging-associated lymphatic obstruction restricts inflammatory clearance and prolongs the inflammatory phase of fracture healing, establishing a link between drainage failure and structural bone deterioration (38, 88).

### 4.3 VEGFR-3-dependent lymphangiogenesis in bone repair

A synchronized lymphangiogenic response helps rebuild intraosseous lymphatic networks that support inflammation resolution and structural regeneration after injury (17, 42). Newly formed lymphatic vessels restore fluid drainage, facilitate inflammatory mediator clearance, and promote a microenvironment conducive to bone repair (38, 42). Therefore, efficient VEGF-C/VEGFR-3 signaling is important for repair-associated lymphangiogenesis.

With aging, VEGF-C/VEGFR-3 signaling becomes progressively impaired, limiting repair-associated lymphangiogenesis (29, 93). In addition, senescent LECs show reduced VEGFR-3 expression, which limits cell proliferation and weakens lymphatic remodeling during repair (29, 79, 93, 94). As the lymphangiogenic response declines, interosseous lymphatics remain poorly organized and functionally insufficient within the injured bone environment (95).

Clinical and experimental evidence further links impaired VEGFR-3 signaling with skeletal deterioration. Impaired VEGF-C/VEGFR-3 responsiveness has been implicated in experimental models of osteolysis, fracture repair, and heterotopic ossification, but direct evidence in human osteoporosis remains limited (42, 87). In experimental injury models, VEGF-C-driven lymphangiogenesis expands intraosseous vessels and promotes inflammatory resolution during bone repair (42). In the intraosseous injury model, VEGF-C/VEGFR-3 activation induced Src phosphorylation and CXCL12 secretion, promoting expansion of Myh11^+^CXCR4^+^ pericytes during skeletal regeneration (17). Independent evidence also shows that VEGF-C helps preserve bone marrow perivascular niche integrity in mice (87). However, the specific VEGF-C/VEGFR-3–CXCL12–Myh11^+^CXCR4^+^ pericyte axis remains mainly supported by the Biswas et al. preclinical model.

Experimental therapies further support a role for VEGFR-3-dependent lymphangiogenesis during skeletal repair. Pharmacological activation of VEGFR-3 restores lymphatic networks in aged bone, thereby reducing osteoclast-driven resorption during fracture repair (42). In aged models, JAK inhibition improves VEGFR-3 responsiveness and enhances lymphatic remodeling capacity. Conversely, inhibition of the IL-6-VEGFR-3 axis (e.g., with SAR131675) impairs lymphatic growth and accelerates bone loss (42). These findings underscore the importance of VEGFR-3-dependent lymphangiogenesis during skeletal repair.

However, the biological effects of VEGF-C/VEGFR-3-dependent lymphangiogenesis are context dependent. Transient activation of this pathway supports repair-associated lymphangiogenesis during skeletal regeneration, whereas persistent or dysregulated activation may promote pathological lymphangiogenesis under disease conditions, as discussed in Sections 5.3 and 6.2. Although these context-dependent effects are supported by preclinical models, their relevance to human skeletal aging remains to be established.

## 5 Clinical symptoms of lymphatic-bone disease

**Figure 4:**
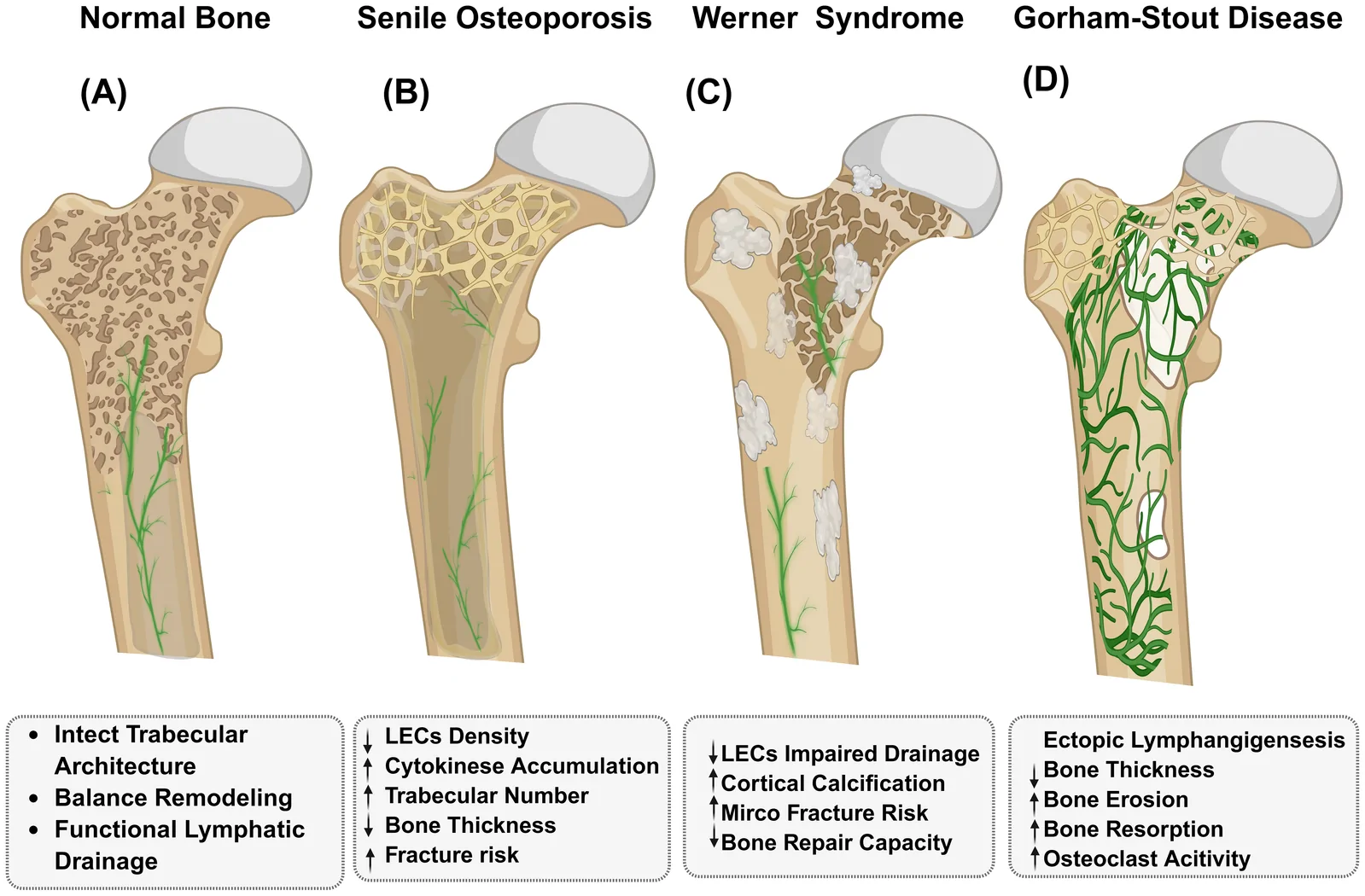
Clinical manifestations of lymphatic-bone aging across distinct pathological contexts. **(A)** Normal bone is shown as a reference state with intact trabecular architecture and balanced bone remodeling. The presence of a broadly distributed functional intraosseous lymphatic network in normal bone remains under investigation. **(B)** In Senile osteoporosis, reduced lymphatic endothelial cell (LEC) density promotes cytokine accumulation, reduces trabecular number and bone thickness, and increases fracture risk. **(C)** In Werner syndrome, LEC dysfunction impairs lymphatic drainage, contributing to cortical calcification, bone fragility, microfracture risk, and reduced skeletal repair capacity. **(D)** In Gorham-Stout disease, ectopic lymphangiogenesis promotes aggressive bone invasion and erosion, increases osteoclastogenesis and fracture risk, and severely disrupts bone thickness and regenerative responses. Created in BioRender. Ahmed, N. (2026) https://BioRender.com/vourafr.

The skeletal consequences of aging extend beyond bone cells and involve the lymphatic system. Age-related remodeling of lymphatic vessels sustains perivascular inflammation, thereby accelerating osteolysis and cortical bone loss (20). Clinical observations support these findings, as diminished lymphatic clearance in elderly fracture patients correlates with delayed healing and, in severe cases, fracture non-union (96). Rare disorders further highlight the skeletal consequences of lymphatic dysfunction. Calcification of lymphatic vessels in progeroid conditions such as Werner syndrome is linked to oxidative stress and vascular dysfunction (10, 97). Inflammatory mediators such as IL-6 enhance RANKL-dependent osteoclastogenesis and contribute to bone loss (98). Similarly, abnormal lymphangiogenesis promotes progressive osteolysis in Gorham-Stout disease (19, 99). Collectively, these examples link lymphatic dysfunction to skeletal decline across age-related and disease-specific skeletal contexts. The distinct clinical manifestations of lymphatic dysfunction across these pathologies are summarized in **Figure 4**.

### 5.1 Senile osteoporosis: age-driven lymphatic vascular decline in bone

Senile osteoporosis involves progressive bone loss, significantly increasing fracture risk in the elderly (100). Lymphatic vessels contribute to skeletal homeostasis by maintaining tissue fluid drainage and inflammatory balance within bone. In aged bone, reduced lymphatic vessel density impairs lymphatic drainage, leading to the accumulation of interstitial inflammatory mediators. Their retention promotes osteoclast activity and disrupts the balance of bone remodeling (20, 30, 48).

Persistent inflammatory signaling further amplifies bone resorption in aged bone. Elevated IL-6 and RANKL levels within the marrow microenvironment stimulate osteoclast precursor differentiation and enhance bone-resorbing activity (98). Clinical imaging studies further support these mechanisms. Lymphoscintigraphy shows reduced inguinal lymph node area, along with reduced visualization of deep lymphatics and popliteal nodes, in patients with non-healing fractures (96).

The molecular basis for this includes communication between the lymphatic and skeletal systems. Downregulation of FOXC2 and VEGF-C disrupts lymphatic barrier integrity and weakens signaling pathways that maintain skeletal homeostasis (101). Concurrently, immune aging expands CD28^−^ T-cell subsets and alters lymphatic chemokine profiles. These changes are associated with osteoclast activation, lower BMD, and increased fracture risk (48, 86).

Clinical observations validate the link between immune imbalance and skeletal decline. CD8^+^ T cell depletion correlates with reduced femoral neck BMD in elderly individuals, supporting the value of lymphocyte profiling in senile osteoporosis (48). Similarly, a high neutrophil-lymphocyte ratio (NLR) in postmenopausal women correlates with declining BMD, supporting its exploratory value as a biomarker (88).

Experimental therapies highlight the importance of lymphatic function in skeletal health. VEGF-C-based lymphatic activation improves lymphatic drainage and reduces bone loss in preclinical models, supporting a contributory role for lymphatic dysfunction in skeletal deterioration (42, 72). These findings support lymphatic involvement in senile osteoporosis; however, direct evidence that LEC senescence is the primary driver of bone loss remains limited.

### 5.2 Werner syndrome: premature lymphatic bone aging

Werner syndrome (WS) is a genetic disorder that particularly affects tissue repair with age, driving segmental progeria and premature skeletal fragility (102). In WS patients, accelerated cellular senescence is associated with lymphatic endothelial dysfunction and altered lymphatic vessel structure (103, 104). The lymphatic system undergoes progressive calcification and elastin degradation. These structural changes reduce vessel contractility and impair lymphatic drainage (10, 105, 106).

Impaired lymphatic transport alters the skeletal microenvironment. Age-related changes in lymphatic vessel stiffness may increase bone brittleness and affect osteocyte network integrity, thereby contributing to osteoclast activation (27, 107). Lymphatic calcification has been proposed to precede trabecular thinning and micro-fractures, but longitudinal human data are lacking (10, 97). Oxidative stress further amplifies this condition. WRN deficiency increases mitochondrial ROS and vascular dysfunction, supporting inflammatory signaling and osteoclast survival (10, 97). ROS activate NF-κB signaling and increase the secretion of proinflammatory cytokines, such as TNF-α and IL-6. These cytokines amplify inflammatory signaling and stimulate RANKL-dependent osteoclastogenesis, thereby promoting bone resorption (31). Thus, WS provides a model of accelerated vascular and lymphatic aging, but it should not be interpreted as direct evidence that LEC senescence drives common age-related osteoporosis.

Immune infiltration within calcified lymphatic vessels further disrupts skeletal balance. Elevated RANKL/OPG ratios enhance osteoclast activity and accelerate mineral loss in affected bone tissues (108). Similar RANKL/OPG imbalances are observed in postmenopausal women with vascular stiffness and bone loss, highlighting the importance of immune-lymphatic interactions in skeletal aging (109, 110).

Therapeutic approaches for WS remain under investigation. ROS scavengers (e.g., NAC) and NF-κB inhibitors show promise but require testing in WS models. It is unknown whether reversing lymphatic calcification can restore bone health. Improving lymphatic function may be a strategy to delay skeletal fragility in Werner syndrome (102).

### 5.3 Gorham-Stout disease: pathological lymphangiogenesis and osteolysis

Gorham-Stout disease (GSD) is a rare disorder characterized by progressive bone loss and abnormal lymphatic vessel formation within bone (19). Most cases present in children or young adults, but rare elderly-onset cases confirm its role as a lifelong lymphatic bone pathology (19, 111). Histological analyses reveal thin, disorganized lymphatic vessels that lack adequate structural support, thereby promoting lymphoedema and degrading the bone matrix (112). These aberrant lymphatic structures are found in areas with high numbers of bone-resorbing cells (osteoclasts) and chronic inflammation (112). In both human and mouse models, osteoclast numbers increase nearly 2.5-fold at sites where lymphatic vessels expand, leading to severe osteolysis (37).

Genetic studies indicate that somatic PIK3CA mutations activate PI3K-AKT-mTOR signaling and drive uncontrolled proliferation of Prox1^+^ and LYVE-1^+^ LECs, leading to intraosseous lymphatic malformation (101). In mutant mouse models, this aberrant lymphatic expansion is accompanied by progressive osteolysis, supporting a role for lymphatic malformation in bone destruction (101). Aberrant LECs also secrete osteoclast-stimulating factors, such as M-CSF, and create a hypoxic, inflammatory microenvironment that favors osteoclast differentiation (37). Unlike the transient repair-associated lymphatic remodeling described in a preclinical bone injury model by Biswas et al. (17), GSD is characterized by sustained and disorganized lymphatic proliferation within an osteoclastogenic inflammatory environment.

VEGF-C/VEGFR-3 signaling further amplifies pathological lymphangiogenesis within bone. Increased VEGF-C signaling stimulates abnormal lymphatic vessel growth and accelerates bone destruction in experimental models (113). These lymphatic expansions promote the accumulation of protein-rich fluid, which degrades bone matrix and recruits inflammatory immune cells, creating a self-reinforcing inflammatory loop that amplifies bone loss (114). Elevated inflammatory cytokines (TNF-α, IL-6, IL-1β) further stimulate osteoclasts and promote lymphatic tissue invasion of bone (111). Interestingly, these inflammatory features partially overlap with the senescence-associated inflammatory environment described during lymphatic vascular aging, suggesting potential mechanistic convergence between chronic inflammation and lymphatic dysfunction (26).

Although GSD is not primarily an age-related disease, it provides a pathological example of dysregulated lymphangiogenesis within bone. Collectively, these findings indicate that the skeletal effects of VEGF-C/VEGFR-3-dependent lymphangiogenesis are context dependent. During aging, impaired VEGF-C/VEGFR-3 responsiveness limits repair-associated lymphangiogenesis and may compromise inflammatory resolution and skeletal regeneration. By contrast, persistent VEGF-C/VEGFR-3 activation in GSD drives uncontrolled lymphatic proliferation, osteoclast activation, and progressive osteolysis. Thus, both insufficient and excessive VEGF-C/VEGFR-3-dependent lymphangiogenesis can disrupt skeletal homeostasis through distinct mechanisms. While these context-dependent effects are supported by preclinical models, their relevance to human skeletal aging remains to be established.

### 5.4 Clinical manifestations and diagnostic evidence of lymphatic-bone dysfunction

Clinical evidence links lymphatic dysfunction to skeletal fragility across several disease settings. These conditions share three features: impaired drainage, abnormal lymphatic remodeling, and inflammatory activation (5, 20). However, their skeletal manifestations differ.

In common senile osteoporosis, lymphatic dysfunction is characterized by progressive lymphatic decline, inflammatory retention, immune imbalance, and impaired fracture repair (36, 96). In Werner syndrome, premature lymphatic calcification and vessel stiffening provide a model of accelerated vascular aging, but this condition does not fully reproduce chronological osteoporosis (10, 97, 102). In Gorham-Stout disease, the dominant feature is pathological intraosseous lymphangiogenesis with progressive osteolysis, often linked to PI3K/AKT/mTOR signaling (19, 101, 111). In rheumatoid arthritis, osteoarthritis, and periodontitis, lymphatic remodeling occurs in chronically inflamed tissues, with loss of mature collecting vessels and expansion of LYVE1^+^ capillary-like lymphatics (72, 115, 116). Secondary lymphatic injury, including radiation-induced lymphatic remodeling and cancer-related lymphedema, further supports a link between impaired lymphatic transport and regional bone loss (17, 117, 118).

Diagnostic evidence also differs by context. Lymphoscintigraphy suggests impaired superficial drainage in delayed or non-healing fractures, with compensatory visualization of deep lymphatics and popliteal nodes (96). Near-infrared ICG imaging links persistent tracer retention to impaired callus formation and cortical porosity in preclinical models (38, 93). Circulating biomarkers, including NLR, CD28^−^ T-cell subsets, and CCL19/CCL21, may help define inflammatory and osteoimmune phenotypes, but they are not specific markers of lymphatic bone disease (40, 42, 48, 84, 86, 88).

Thus, these disorders support a clinically relevant lymphatic-bone axis, but they represent different biological contexts. Prospective studies should combine lymphatic imaging, BMD, fracture outcomes, and inflammatory biomarkers to define which lymphatic changes are causal, compensatory, or secondary to tissue injury.

## 6 Translational perspectives and potential therapeutic approaches

Lymphatic vascular senescence provides a therapeutic entry point for skeletal aging (17, 20, 26). Unlike conventional antiresorptive interventions, lymphatic-targeted strategies address the inflammatory basis of osteoporosis (21, 26, 49). These approaches range from drug repurposing to biologics supported by preclinical and clinical evidence (49, 91, 99, 119). Most therapeutic evidence remains preclinical or disease-specific, and direct clinical validation in common age-related osteoporosis is still lacking. **Table 1** summarizes representative candidate therapies, their mechanisms, and their current level of evidence.

**Table 1:** Candidate interventions targeting lymphatic dysfunction-associated skeletal changes.

Therapeutic agent	Proposed mechanism for bone-lymphatic axis	Current clinical status	Translational outcome	Evidence/key limitation	References
Ruxolitinib	JAK inhibition suppresses SASP and restores VEGF-C responsiveness in aged lymphatics	FDA-approved (myelofibrosis)	Reduced osteolysis and improved lymphatic function in aged osteolysis models	Preclinical bone evidence; immune-related adverse effects	(42)
Tocilizumab (Anti-IL-6)	IL-6 receptor blockade suppresses osteoclastogenic signaling	FDA-approved (RA)	Reduced bone resorption markers and preserved femoral neck BMD in inflammatory bone loss	Evidence primarily derived from RA; lymphatic endpoints not assessed	(119)
Clopidogrel	P2Y12 platelet inhibition reduces lymphatic thrombosis	FDA-approved (cardiovascular)	Restores lymph flow, enhances drainage, and improves fracture healing	Fracture repair model; bleeding risk and timing concerns	(38, 92)
Sirolimus	mTOR inhibition suppresses pathological lymphangiogenesis	FDA-approved (Various)	Stabilizes lymphatic malformations and reduces osteolysis in Gorham-Stout disease	GSD/GLA evidence; relevance to common osteoporosis remains uncertain	(99)
Senolytics (Dasatinib + Quercetin)	Clears senescent cells and reduces SASP signaling	Preclinical	Improved fracture healing in aged mice	Aged fracture model; lymphatic-specific effects remain unclear	(120, 121)

These agents target inflammatory, lymphatic, platelet, mTOR, and senescence-associated pathways. Current evidence supports their relevance in selected preclinical or disease-specific contexts, whereas direct clinical evidence in common age-related osteoporosis remains limited.

### 6.1 Targeting inflammation and senescence

Promising therapeutic strategies focus on suppressing the proinflammatory SASP (6, 21, 45). Interleukin-6 (IL-6) receptor blockade with tocilizumab demonstrates efficacy in ACPA-positive rheumatoid arthritis patients, reducing bone resorption markers and restoring BMD (49, 119). Similarly, JAK inhibition with ruxolitinib reduces systemic inflammation and restores VEGFR-3 signaling in aged LECs (42, 91, 122–124). Although tocilizumab and ruxolitinib are approved for other indications, their application to skeletal aging remains experimental. Existing evidence supports further evaluation in defined high-risk populations, with careful attention to treatment timing, skeletal endpoints, and immune-related adverse effects (49, 119, 123).

### 6.2 Reestablishment of lymphatic vascular function

Restoring VEGF-C/VEGFR-3 signaling improves lymphatic function in preclinical bone models, but its skeletal effects differ by inflammatory state, VEGFR-3 responsiveness, and signal strength (42, 92, 122). In adult inflammatory osteolysis models, recombinant VEGF-C promoted intraosseous lymphatic expansion, reduced inflammatory cell infiltration, and decreased osteoclast activity, thereby limiting osteolysis (42). In aged mice, VEGF-C failed to restore this response because senescent and adipogenic marrow stromal cells suppressed LEC responsiveness; ruxolitinib reduced this SASP-related inhibition and restored lymphatic responsiveness to VEGF-C (42). In VEGF-C overexpression models, sustained local VEGF-C signaling induced bone lymphatics, increased osteoclast number and activity, and caused cortical porosity and trabecular weakening (113). Together, these findings highlight that therapeutic modulation of VEGF-C/VEGFR-3 signaling requires precise regulation to restore physiological lymphatic function while avoiding pathological lymphatic expansion.

Conversely, mTOR inhibition with sirolimus has shown clinical benefit in generalized lymphatic anomaly and Gorham-Stout disease, including patients with bone involvement, providing proof-of-concept for targeting pathological lymphangiogenesis (99). Another therapeutic direction is to restore the supportive function of lymphatic-associated marrow niches. Biswas et al. showed in a preclinical intraosseous injury model that LEC-derived CXCL12 promotes expansion of Myh11^+^CXCR4^+^ pericytes during skeletal regeneration (17). This lymphatic–pericyte response is impaired in aged animals following genotoxic stress, suggesting impaired CXCL12-dependent niche support during bone regeneration (87). VEGF-C delivery after irradiation restores endothelial integrity and LepR^+^ niche architecture, supporting recovery of the bone marrow perivascular niche (87). This CXCL12–pericyte mechanism is primarily supported by preclinical data on intraosseous injury.

Lymphatic platelet thrombosis (LPT) is another barrier to fracture repair. In preclinical fracture models, the P2Y12 inhibitor clopidogrel reduced lymphatic platelet thrombosis, restored lymphatic drainage, and improved fracture healing (38, 92). Short-term senolytic treatment with dasatinib plus quercetin reduced the burden of callus senescent cells and improved fracture repair in aged mice (120). Aged callus senescent cells have also been shown to impair fracture healing through TGF-β1-mediated mechanisms (121). However, the bone-lymphatic effects of dasatinib plus quercetin remain incompletely defined. Furthermore, several experimental strategies have demonstrated proof of concept in preclinical models, as summarized in **Table 2**.

**Table 2:** Experimental approaches targeting the bone-lymphatic axis in preclinical models.

Therapeutic approach	Proposed mechanism for bone-lymphatic axis	Experimental model	Translational outcome	Evidence/key limitation	References
Recombinant VEGF-C	VEGFR-3 activation enhances lymphatic responsiveness	Adult calvarial osteolysis model	Reduced osteolysis in adult mice; the effect was age-dependent	Age-dependent efficacy limits its use as monotherapy	(42)
VEGF-C + ruxolitinib	Dual targeting of VEGFR-3 signaling and SASP	Aged osteolysis model	Increases lymphatic density, reduces SASP signaling and osteolysis, restores lymphatic responsiveness	The combination has not been tested clinically	(42)
FGF9 therapy	Activates FGFR3-BMPR1a-Smad1/5 signaling in LECs	Acquired heterotopic ossification model	Enhances lymphatic vessel formation, reduces joint damage, and inhibits heterotopic ossification	Its relevance to aging-related fracture or osteoporosis remains unclear	(125)
Anti-PDPN neutralizing antibody	Blocks the CLEC-2/PDPN axis and reduces lymphatic platelet thrombosis	Fracture mouse model	Reduces lymphatic platelet thrombosis, improves lymphatic drainage, and enhances fracture repair, with increased callus BV/TV and trabecular thickness	Clinical safety of platelet–lymphatic targeting remains untested	(38)
Local AAV-VEGFC delivery	Increases the collecting lymphatic vessels and improves local lymphatic drainage	Inflammatory periodontitis model	Reduced alveolar bone loss, TRAP-positive osteoclast surface, and improved local bone indices	Its relevance to skeletal aging remains untested	(72)
SAR131675 (mechanistic inhibitor)	VEGFR-3 tyrosine kinase blockade	Tibial fracture model	Reduces ectopic lymphangiogenesis and decreases bone porosity; may impair regeneration	VEGFR-3 blockade may impair bone repair	(42)

These strategies provide proof-of-concept for modulating lymphatic remodeling, drainage, and repair-associated signaling, but their relevance to human skeletal aging requires further validation.

### 6.3 Biomarkers and clinical translation

Serum CCL19/21 levels rise with age, and CCR7 signaling promotes osteoclast precursor migration, supporting their exploratory value as candidate biomarkers (40, 48, 84). Elevated NLR correlates with lower BMD in elderly cohorts, indicating an exploratory value for patient stratification (42, 88). Lymphatic imaging may assist biomarker discovery, but prospective validation is required (96). Advanced lymphatic imaging techniques, including lymphoscintigraphy, provide functional endpoints for future clinical studies.

IL-6 blockade and JAK inhibition are supported by pharmacological evidence from defined disease contexts: tocilizumab reduced bone resorption and increased femoral BMD in ACPA-positive rheumatoid arthritis, whereas JAK inhibition preserved bone integrity and restored lymphatic responsiveness in aged and progeria models (42, 49, 119, 123). VEGF-C therapy requires safety optimization for bone applications because its skeletal effects depend on timing, dose, and tissue context (113). Low-dose clopidogrel supports preclinical targeting of lymphatic platelet thrombosis in fracture repair; clinical translation requires further definition of bleeding risk, perioperative timing, and lymphatic imaging endpoints (38, 122). Early-phase studies should integrate lymphatic imaging with skeletal endpoints, including BMD, callus formation, fracture healing time, and bone turnover markers, to determine whether restoring lymphatic function can improve skeletal repair in aging populations (96). The convergence of preclinical skeletal models with selected clinical experience in lymphatic disorders supports further investigation of these therapeutic strategies (99, 126).

## 7 Conclusion and future perspectives

Age-related lymphatic dysfunction is a plausible contributor to skeletal degeneration through effects on drainage, inflammatory retention, and osteoimmune regulation. However, the current evidence remains uneven. Direct evidence for functional intraosseous lymphatics derives mainly from preclinical injury and osteolysis models, whereas their distribution and physiological roles during common human skeletal aging remain unresolved. Findings from non-healing fractures, inflammatory bone diseases, Werner syndrome, and Gorham-Stout disease provide additional mechanistic context but do not establish a unified local lymphatic mechanism in common osteoporosis.

Available models further indicate that lymphangiogenesis has context-dependent effects. Regulated, repair-associated lymphatic expansion can support inflammatory resolution and tissue repair in defined models, whereas sustained, disorganized, or mutation-driven lymphatic expansion can promote osteoclastogenesis and osteolysis. Thus, aging-associated lymphatic regression and pathological lymphatic overgrowth represent distinct disruptions of regulated lymphatic homeostasis. Future studies should determine whether restoring lymphatic function can improve skeletal repair while preserving physiological lymphatic regulation.

Future studies should establish lymphatic dysfunction as a measurable feature of bone aging. Longitudinal studies should combine lymphatic imaging, BMD, fracture outcomes, bone turnover markers, and inflammatory profiles to determine whether lymphatic impairment precedes bone loss, accompanies established skeletal degeneration, or reflects impaired repair capacity. Functional endpoints will be essential. Vessel density alone is insufficient; drainage capacity, lymphatic contractility, inflammatory clearance, and responsiveness to VEGF-C/VEGFR-3 provide more direct measures of the lymphatic contribution to skeletal pathology.

Therapeutic studies should follow the same evidence hierarchy. JAK/IL-6 inhibition targets SASP-associated inflammatory signaling; VEGF-C-based approaches restore lymphatic responsiveness; P2Y12 inhibition addresses lymphatic platelet thrombosis during fracture repair; and senolytics reduce the senescence-associated inflammatory burden. These strategies are supported by mechanistic evidence, but most data remain preclinical or disease-specific. Translation to older adults will require well-defined patient groups, treatment windows, skeletal endpoints, and safety monitoring, particularly for immune modulation and antiplatelet therapy. If validated in human studies, lymphatic-targeted interventions could complement antiresorptive and anabolic therapies by improving inflammatory clearance and repair capacity in aging bone.
